# Supplementary material for: A live imaging system to analyze spatiotemporal dynamics of RNA polymerase II modification in Arabidopsis thaliana
Source: Commun Biol. 2021 May 14;4:580. doi: 10.1038/s42003-021-02106-0 (PMC8121908; doi:10.1038/s42003-021-02106-0)
Supplement: Supplementary file 2 — Supplementary Information [file 42003_2021_2106_MOESM2_ESM.pdf]

Supplementary materials

"A live imaging system to analyze spatiotemporal dynamics of RNA polymerase II modification in *Arabidopsis thaliana*"

Mio K. Shibuta[1], Takuya Sakamoto[2], Tamako Yamaoka[2], Mayu Yoshikawa[2], Shusuke Kasamatsu[3], Noriyoshi Yagi[2], Satoru Fujimoto[2], Takamasa Suzuki[4], Satoshi Uchino[5], Yuko Sato[5,6], Hiroshi Kimura[6], and Sachihiro Matsunaga[1]

[1] Graduate School of Frontier Sciences, Department of Integrated Biosciences, The University of Tokyo, 5-1-5 Kashiwanoha, Kashiwa, Chiba, 277-8562, Japan

[2]Faculty of Science and Technology, Department of Applied Biological Science, Tokyo University of Science, 2641 Yamazaki, Noda, Chiba, 278-8510, Japan

[3]Academic Assembly (Faculty of Science), Yamagata University, 1-4-12 Kojirakawa, Yamagata-shi, Yamagata 990-8560, Japan

[4]College of Bioscience and Biotechnology, Chubu University, 1200 Matsumoto-cho, Kasugai, Aichi, 487-8501, Japan

[5]Graduate School of Bioscience and Biotechnology, Tokyo Institute of Technology, 4259 Nagatsuta, Midori-ku, Yokohama, 226-8501, Japan

[6]Cell Biology Center, Institute of Innovative Research, Tokyo Institute of Technology, 4259 Nagatsuta, Midori-ku, Yokohama, 226-8503, Japan

This file includes: Supplementary Fig. 1-6

Other Supplementary Materials for this manuscript include the following: Supplementary Data 1-7

Supplementary Fig. 1

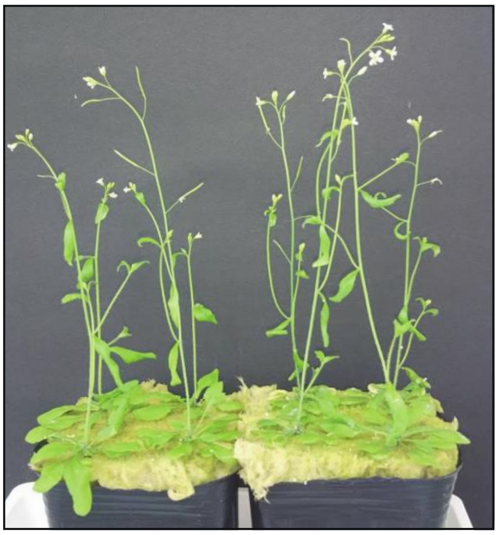

Supplementary Fig. 1: Growth phenotype of Ser2P-mintbody-expressing seedling.

Growth phenotypes of wild-type (WT) and *pUBQ10:Ser2P-mintbody* seedlings grown under long days.

Supplementary Fig. 2

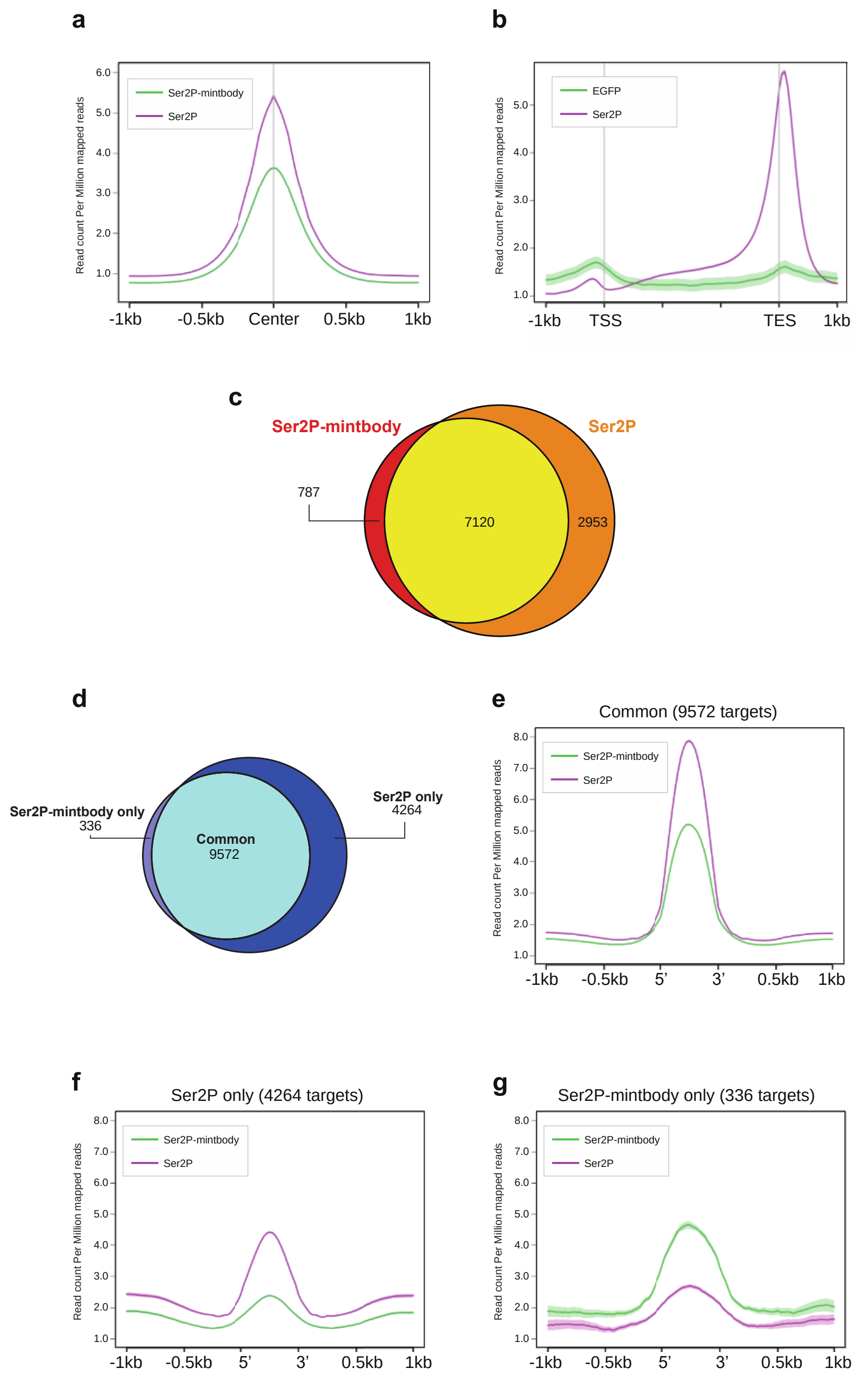

Supplementary Fig. 2: Average profile plot showing that Ser2P-mintbody and RNAPII Ser2P have a similar distribution profile and localize to common loci.

(a) Distribution of Ser2P-mintbody around the peak summits of RNAPII Ser2P target genes in *pUBQ10:Ser2P-mintbody* seedlings. Center: peak summit of RNAPII Ser2P; −1 kb, −0.5 kb: 1 or 0.5 kb upstream of Center; 0.5 kb, 1 kb: 0.5 or 1 kb downstream of Center. (b) Distribution of EGFP-3xFLAG and Ser2P vs gene-body shown as an

average profile plot. TSS: transcription start site; TES: transcription end site; −1 kb: 1 kb upstream of TSS; 1 kb: 1 kb downstream of TES. (c) Venn diagram showing the overlap of Ser2P-mintbody- and Ser2P-enriched genes of another biological replicate (rep2). (d) Venn diagram of peak groups. (e–g) Distribution of Ser2P-mintbody and RNAPII Ser2P around peak summits in *pUBQ10:Ser2P-mintbody* seedlings. 5′: start point of peak summit; 3′: end point of peak summit; −1 kb, −0.5 kb: 1 or 0.5 kb upstream of 5′; 0.5 kb, 1 kb: 0.5 or 1 kb downstream of 3′.

Supplementary Fig. 3

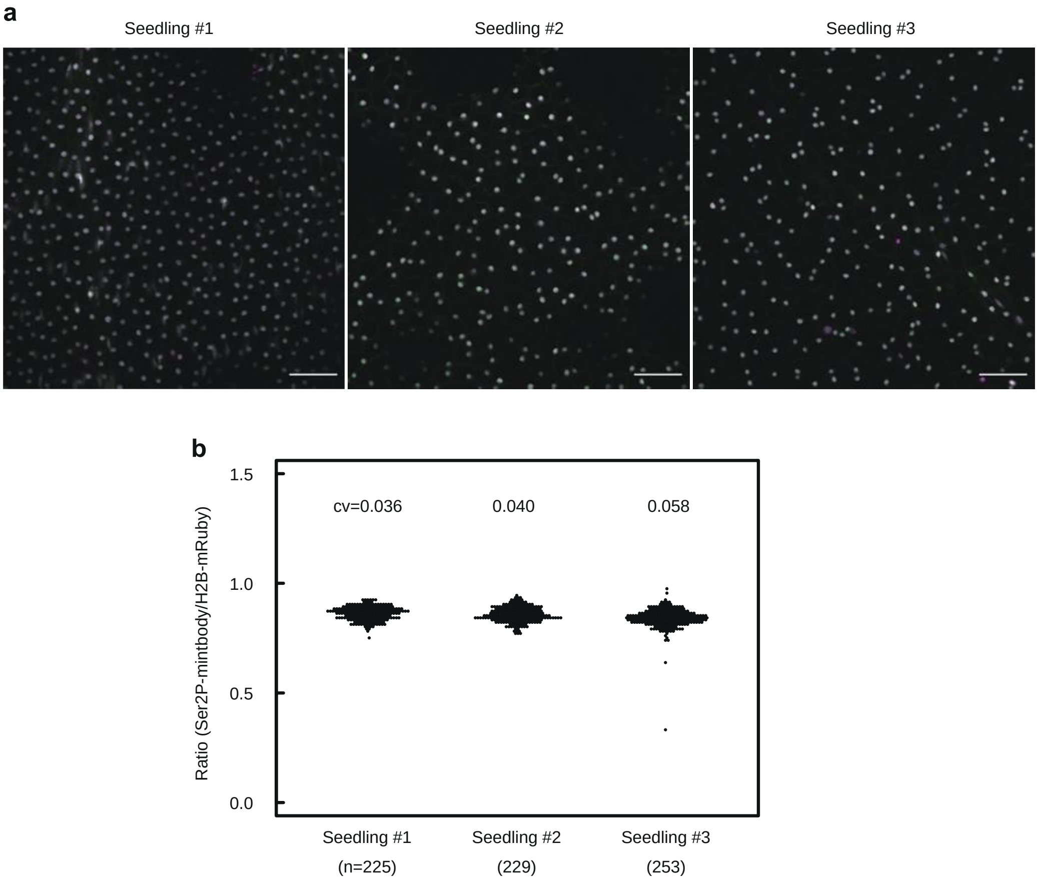

Supplementary Fig. 3: Two-component system in *A. thaliana*.

(a) Observations with the two-component system in seedlings expressing *pRPS5a:Ser2P-mintbody-IntF2A-H2B-mRuby*. Petal epidermal cells were observed in three T_2 seedlings raised from the same T_1 seedling. Merged images of Ser2P-mintbody (green) and H2B-mRuby (magenta) are shown. Scale bars, 30 µm. (b) Ratios of the fluorescence intensity of Ser2P-mintbody relative to H2B-mRuby. Ratios were calculated from 225–253 nuclei in each petal, and values are shown in dot plots. n=biologically independent cells. CV: coefficient of variation. Source data underlying the plot are available as Supplementary Data 6.

Supplementary Fig. 4

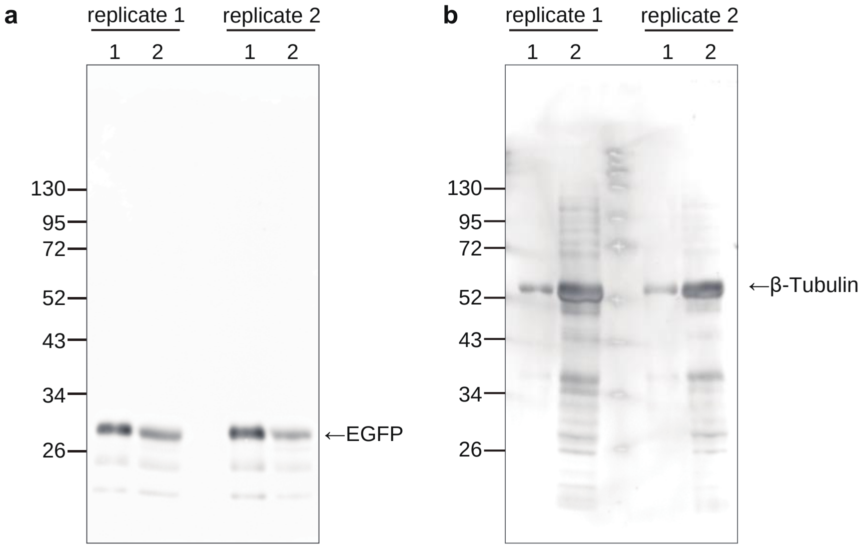

Supplementary Fig. 4: Uncropped images of western blots in Fig. 2c.

(a, b) Uncropped images from Fig. 2c of membranes processed with anti-GFP antibody (a) or anti-β-tubulin antibody as a loading control (b). Uncropped images of replicate 2 in Fig. 2c and a biological replicate are shown. Lane 1: sample extracted from a plant expressing *p35S:EGFP*; lane 2: sample extracted from a plant expressing *p35S:EGFP-IntF2A-tdTomato-NLS*. The membrane was first processed with anti-GFP antibody and, after stripping, was reprobed with anti-β-tubulin antibody.

Supplementary Fig. 5

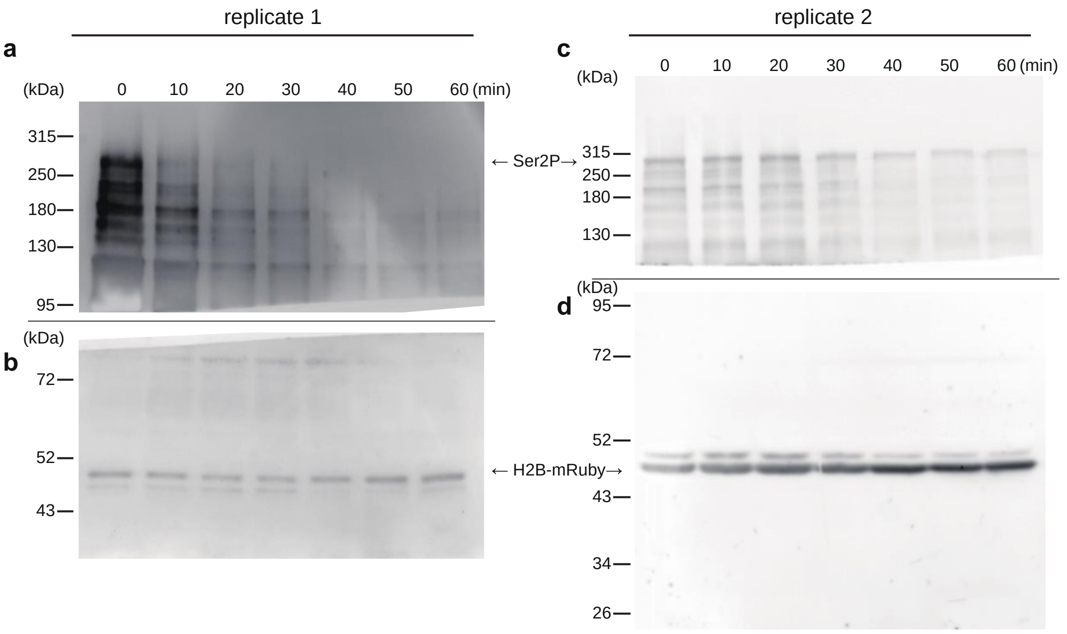

Supplementary Fig. 5: Uncropped images of western blots in Fig. 3b.

(a-d) Uncropped images from Fig. 3b of membranes processed with anti-Ser2P antibody (MABI0602) (a, c) or anti-RFP antibody as a loading control (b, d). Uncropped images of replicate 1 in Fig. 3b and a biological replicate are shown. After blotting, the membranes were cut and divided into the upper part (a, c) and the lower part (b, d). The upper and lower parts were processed with anti-Ser2P antibody (MABI0602) and anti-RFP antibody, respectively.

Supplementary Fig. 6

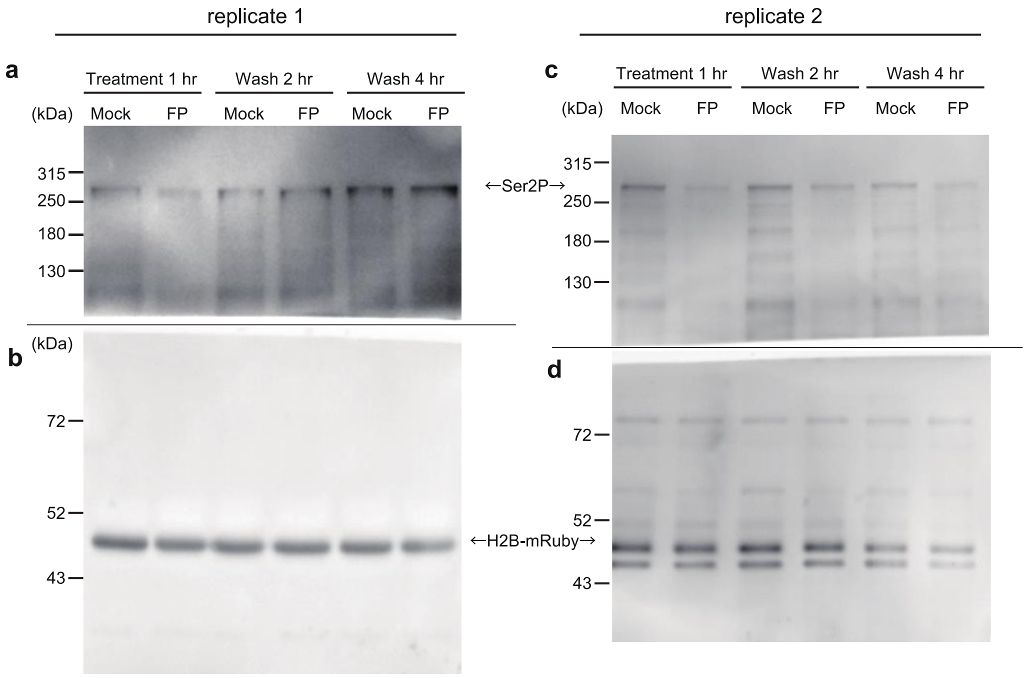

Supplementary Fig. 6: Uncropped images of western blots in Fig. 3e.

(a–d) Uncropped images from Fig. 3e of membranes processed with anti-Ser2P antibody (MABI0602) (a, c) or anti-RFP antibody as a loading control (b, d). Uncropped images of replicate 1 in Fig. 3e and a biological replicate are shown. After blotting, the membranes were cut and divided into the upper part (a, c) and the lower part (b, d). The upper and lower parts were processed with anti-Ser2P antibody (MABI0602) and anti-RFP antibody, respectively. FP: flavopiridol.
