# Supplementary material for: A live imaging system to analyze spatiotemporal dynamics of RNA polymerase II modification in Arabidopsis thaliana
Source: Commun Biol. 2021 May 14;4:580. doi: 10.1038/s42003-021-02106-0 (PMC8121908; doi:10.1038/s42003-021-02106-0)
Supplement: Supplementary file 3 — Reporting Summary [file 42003_2021_2106_MOESM3_ESM.pdf]

nature research

Corresponding author(s): Sachihiro Matsunaga

Last updated by author(s): Mar 23, 2021

Reporting Summary

Nature Research wishes to improve the reproducibility of the work that we publish. This form provides structure for consistency and transparency in reporting. For further information on Nature Research policies, see our Editorial Policies and the Editorial Policy Checklist.

Statistics

For all statistical analyses, confirm that the following items are present in the figure legend, table legend, main text, or Methods section.

n/a	Confirmed	
☐	☒	The exact sample size (*n*) for each experimental group/condition, given as a discrete number and unit of measurement
☐	☒	A statement on whether measurements were taken from distinct samples or whether the same sample was measured repeatedly
☐	☒	The statistical test(s) used AND whether they are one- or two-sided<br>*Only common tests should be described solely by name; describe more complex techniques in the Methods section.*
☒	☐	A description of all covariates tested
☒	☐	A description of any assumptions or corrections, such as tests of normality and adjustment for multiple comparisons
☐	☒	A full description of the statistical parameters including central tendency (e.g. means) or other basic estimates (e.g. regression coefficient) AND variation (e.g. standard deviation) or associated estimates of uncertainty (e.g. confidence intervals)
☐	☒	For null hypothesis testing, the test statistic (e.g. *F*, *t*, *r*) with confidence intervals, effect sizes, degrees of freedom and *P* value noted<br>*Give P values as exact values whenever suitable.*
☒	☐	For Bayesian analysis, information on the choice of priors and Markov chain Monte Carlo settings
☒	☐	For hierarchical and complex designs, identification of the appropriate level for tests and full reporting of outcomes
☒	☐	Estimates of effect sizes (e.g. Cohen's *d*, Pearson's *r*), indicating how they were calculated

Our web collection on statistics for biologists contains articles on many of the points above.

Software and code

Policy information about availability of computer code

Data collection	We used Fiji (https://fiji.sc/) for imaging data and ExPASy (https://www.expasy.org) for molecular weight estimation. We used original code (https://zenodo.org/record/4628571#.YFl3pC3AOgQ) for fluorescence intensity ratio calculation.
Data analysis	We used open souse softwares include: Bowtie2 (version 2.3.5.1), SAMTools (version 1.9), BEDTools (version 2.29.2), MACS2 (version 2.1.1), as well as RStudio version 1.2.5033, and Python to analyze all the datasets.

For manuscripts utilizing custom algorithms or software that are central to the research but not yet described in published literature, software must be made available to editors and reviewers. We strongly encourage code deposition in a community repository (e.g. GitHub). See the Nature Research guidelines for submitting code & software for further information.

Data

Policy information about availability of data

All manuscripts must include a data availability statement. This statement should provide the following information, where applicable:

- Accession codes, unique identifiers, or web links for publicly available datasets
- A list of figures that have associated raw data
- A description of any restrictions on data availability

ChIP-seq data that support the findings of this study have been deposited in the DNA Data Bank of Japan (accession no. DRA011114, http://trace.ddbj.nig.ac.jp/DRASearch/submission?acc=DRA011114).
Original western blotting images were deposited in figshare (DOI: 10.6084/m9.figshare.14265617, https://figshare.com/s/9d02888fc011c5fe0ec8).
The code for fluorescence intensity ratio calculation were deposited in Zenodo. (DOI: 10.5281/zenodo.4628571, https://zenodo.org/record/4628571#.YFl3pC3AOgQ).

Field-specific reporting

Please select the one below that is the best fit for your research. If you are not sure, read the appropriate sections before making your selection.

- [x] Life sciences
- [ ] Behavioural & social sciences
- [ ] Ecological, evolutionary & environmental sciences

For a reference copy of the document with all sections, see nature.com/documents/nr-reporting-summary-flat.pdf

Life sciences study design

All studies must disclose on these points even when the disclosure is negative.

Sample size	No sample-size calculation was performed. Samples size was chosen according to the standard generally accepted in the field of plant cell biology.
Data exclusions	No data were excluded from the analyses.
Replication	Different amount of replicates were adopted dependently by the analysis, in accordance with the standard generally accepted in field of plant cell biology.
Randomization	The plates were randomly placed in the growth chamber to exclude positional effects during growth.
Blinding	No blinding was applied. Because we applied identical settings to all the samples, blinding was not essential.

Reporting for specific materials, systems and methods

We require information from authors about some types of materials, experimental systems and methods used in many studies. Here, indicate whether each material, system or method listed is relevant to your study. If you are not sure if a list item applies to your research, read the appropriate section before selecting a response.

Materials & experimental systems

n/a	Involved in the study	
[ ]	[x]	Antibodies
[x]	[ ]	Eukaryotic cell lines
[x]	[ ]	Palaeontology and archaeology
[x]	[ ]	Animals and other organisms
[x]	[ ]	Human research participants
[x]	[ ]	Clinical data
[x]	[ ]	Dual use research of concern

Methods

n/a	Involved in the study	
[ ]	[x]	ChIP-seq
[x]	[ ]	Flow cytometry
[x]	[ ]	MRI-based neuroimaging

Antibodies

Antibodies used	Mouse monoclonal anti GFP antibodies (11814460001, Roche)<br>Rabbit monoclonal anti RNA polymerase II CTD repeat YSPTSPS (phospho S2) (ab193468, abcam)<br>Rabbit monoclonal anti GFP (ab290, abcam)<br>Mouse monoclonal anti β-tubulin (MAB3408, Sigma-Ardrich)<br>Rabbit Polyclonal anti RFP (R10367, Thermo Fisher SCIENTIFIC)<br>Mouse monoclonal anti RNA polymerase II CTD repeat YSPTSPS (phospho S2) (MABI0602, MAB Institute)<br>Goat polyclonal anti IgG (rabbit) pAb-HRP conjugate (458, MBL)<br>Goat polyclonal anti IgG (mouse) HRP conjugate (W402B, Promega)
Validation	Information about these antibodies are available at the manufacturer's website.<br>https://www.sigmaaldrich.com/catalog/product/roche/11814460001?lang=ja&region=JP<br>https://www.abcam.co.jp/rna-polymerase-ii-ctd-repeat-ysptsps-phospho-s2-antibody-epr18855-ab193468.html<br>https://www.abcam.co.jp/gfp-antibody-ab290.html<br>https://www.emdmillipore.com/US/en/product/Anti-Tubulin-Antibody-beta-clone-KMX-1,MM_NF-MAB3408?bd=1<br>https://www.thermofisher.com/antibody/product/RFP-Antibody-Polyclonal/R10367<br>https://ruo.mbl.co.jp/bio/dtl/A/?pcd=458<br>https://www.promega.jp/products/protein-detection/primary-and-secondary-antibodies/anti_mouse-igg-h-and-l-hrp-conjugate/?catNum=W4021<br>https://labchem-wako.fujifilm.com/jp/product/detail/W01W0101-2238.html

ChIP-seq

Data deposition

☒ Confirm that both raw and final processed data have been deposited in a public database such as GEO.

☒ Confirm that you have deposited or provided access to graph files (e.g. BED files) for the called peaks.

Data access links<br>*May remain private before publication.*	DRA011114, http://trace.ddbj.nig.ac.jp/DRASearch/submission?acc=DRA011114
Files in database submission	Original ChIP-seq read files:<br>pUBQ10:Ser2P-mintbody_anti-GFP_rep1:42B3_GFP_S9_L001_R1_001.fastq.gz,42B3_GFP_S9_L002_R1_001.fastq.gz,42B3_GFP_S9_L003_R1_001.fastq.gz,42B3_GFP_S9_L004_R1_001.fastq.gz; pUBQ10:Ser2P-mintbody_anti-Ser2P_rep1:42B3_Pc26_S10_L001_R1_001.fastq.gz,42B3_Pc26_S10_L002_R1_001.fastq.gz,42B3_Pc26_S10_L003_R1_001.fastq.gz,42B3_Pc26_S10_L004_R1_001.fastq.gz; p35S:EGFP-3xFLAG_anti-GFP_rep1:EGFP_GFP_S12_L001_R1_001.fastq.gz,EGFP_GFP_S12_L002_R1_001.fastq.gz,EGFP_GFP_S12_L003_R1_001.fastq.gz,EGFP_GFP_S12_L004_R1_001.fastq.gz; p35S:EGFP-3xFLAG_anti-Ser2P_rep1:EGFP_Pc26_S13_L001_R1_001.fastq.gz,EGFP_Pc26_S13_L002_R1_001.fastq.gz,EGFP_Pc26_S13_L003_R1_001.fastq.gz,EGFP_Pc26_S13_L004_R1_001.fastq.gz; pUBQ10:Ser2P-mintbody_anti-GFP_rep2:Shibuta_1_S20_L001_R1_001.fastq.gz,Shibuta_1_S20_L002_R1_001.fastq.gz,Shibuta_1_S20_L003_R1_001.fastq.gz,Shibuta_1_S20_L004_R1_001.fastq.gz; pUBQ10:Ser2P-mintbody_anti-Ser2P_rep2:Shibuta_2_S21_L001_R1_001.fastq.gz,Shibuta_2_S21_L002_R1_001.fastq.gz,Shibuta_2_S21_L003_R1_001.fastq.gz,Shibuta_2_S21_L004_R1_001.fastq.gz; p35S:EGFP-3xFLAG_anti-GFP_rep2:Shibuta_3_S24_L001_R1_001.fastq.gz,Shibuta_3_S24_L002_R1_001.fastq.gz,Shibuta_3_S24_L003_R1_001.fastq.gz,Shibuta_3_S24_L004_R1_001.fastq.gz; p35S:EGFP-3xFLAG_anti-Ser2P_rep2:Shibuta_4_S22_L001_R1_001.fastq.gz,Shibuta_4_S22_L002_R1_001.fastq.gz,Shibuta_4_S22_L003_R1_001.fastq.gz,Shibuta_4_S22_L004_R1_001.fastq.gz
Genome browser session<br>(e.g. UCSC)	No longer applicable.

Methodology

Replicates	Two independent biological replicates were analyzed for each target.
Sequencing depth	ChIP-seq was sequenced with about 10 million reads for each sample. Type of run was 75 bp single-end.
Antibodies	Mouse monoclonal anti GFP antibodies (11814460001, Roche), Mouse monoclonal anti RNA polymerase II CTD repeat YSPTSPS (phospho S2) (MABI0602, MAB Institute)
Peak calling parameters	Command line : macs2 callpeak -t ChIP.bam -c control(anti GFP in p35S:EGFP-3xFLAG).bam -f BAM -q 0.05 -g 1.26e8
Data quality	Data quality was controlled with Trimmomatic and FastQC. We also visualized peak signals on genome browser for each dataset.
Software	We used bowtie, SAMtools, bedtools, and MACS2.
